# Changing Cellular Location of CheZ Predicted by Molecular Simulations

**DOI:** 10.1371/journal.pcbi.0020039

**Published:** 2006-04-28

**Authors:** Karen Lipkow

**Affiliations:** Department of Physiology, Development and Neuroscience, University of Cambridge, Cambridge, United Kingdom; Cornell University, United States of America

## Abstract

In the chemotaxis pathway of the bacterium *Escherichia coli,* signals are carried from a cluster of receptors to the flagellar motors by the diffusion of the protein CheY-phosphate (CheYp) through the cytoplasm. A second protein, CheZ, which promotes dephosphorylation of CheYp, partially colocalizes with receptors in the plasma membrane. CheZ is normally dimeric in solution but has been suggested to associate into highly active oligomers in the presence of CheYp. A model is presented here and supported by Brownian dynamics simulations, which accounts for these and other experimental data: A minority component of the receptor cluster (dimers of CheA_short_) nucleates CheZ oligomerization and CheZ molecules move from the cytoplasm to a bound state at the receptor cluster depending on the current level of cellular stimulation. The corresponding simulations suggest that dynamic CheZ localization will sharpen cellular responses to chemoeffectors, increase the range of detectable ligand concentrations, and make adaptation more precise and robust. The localization and activation of CheZ constitute a negative feedback loop that provides a second tier of adaptation to the system. Subtle adjustments of this kind are likely to be found in many other signaling pathways.

## Synopsis

In order to function effectively, a living cell must not only synthesize the correct molecules but also put them in the correct place. Understanding how this positioning occurs, and what its consequences are, is a matter of great interest and concern to contemporary biologists. The author here proposes a novel mechanism that will enhance the ability of a bacterial cell to perform chemotaxis—the ability to swim toward sources of food or away from noxious substances. In this hypothesis, a key protein in the chemotaxis pathway moves dynamically between the membrane and the cytoplasm depending on the presence of attractants or repellents. This idea is explored and tested by means of detailed molecular simulations in which all of the relevant molecules are shown in their correct location in the cell. The simulations show that the proposed shift in location of the key molecule will improve the speed, range, and robustness of the cell's response. It seems likely that similar movements of proteins will occur in many other signaling pathways.

## Introduction

Experimental results of the past several years reveal that the bacterial cytoplasm is more complex and sophisticated than previously thought. To cite a recent review on the prokaryotic cell cycle: “Many signal transduction proteins are dynamically localized to specific subcellular addresses … and proper localization is essential for their function” [1]. The well-studied bacterial chemotaxis pathway is now known to depend on two kinds of large multiprotein complexes: inputs are detected by a cluster of receptors and associated proteins at one end of the cell, while flagellar motors elsewhere in the cell generate the system's output [2,3]. A small protein, CheY, achieves communication between these two complexes by diffusing freely through the cytoplasm. This protein receives its phosphate from the histidine kinase CheA, associated with the inner face of the receptor cluster, at a rate that depends on chemotactic stimulation. From there, phosphorylated CheY (CheYp) diffuses to the four-or-so motors, where it causes a change in rotational switching frequency (i.e., duration of swimming or tumbling behavior) according to its local concentration. The signal is initiated and terminated through the level of the kinase activity. It adapts to constant stimulus levels and returns to its steady-state value through changes in receptor methylation by the enzymes CheR and CheB. The signal is also stopped directly through dephosphorylation of CheYp, which is promoted by the protein CheZ.

The present report adds to this picture by proposing that CheZ is a second molecule of the pathway, which changes its location during the signal transduction process. According to this model, the relocalization coincides with changes in dephosphorylation activity and leads to a second tier of adaptation, by regulating the termination of the signal. Reminiscent of the migration of proteins of the Min system that ensures the correct positioning of the bacterial cell division plane [4], the changing location of CheZ should serve to sharpen responses of the cell to attractants and repellents and make adaptation more precise. The presented proposal is based on published data, supported by quantitative computer simulations, and makes specific predictions that can be tested by experiment.

## Results

### Hypothesis and Biological Background

**Figure 1:**
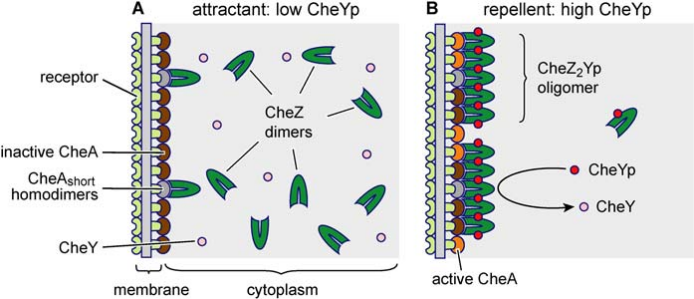
Schematic of the Dynamic CheZ Hypothesis (A) A layer of CheA dimers is positioned at the cytoplasmic face of the polar chemoreceptor cluster. Interspersed with the catalytically active CheA dimers are CheA_short_ homodimers, which act as anchoring points for CheZ dimers. In the absence of CheYp, a condition produced by saturating concentration of attractants, the remaining CheZ dimers diffuse freely in the cytoplasm. (B) Upon increased phosphorylation of CheY, which occurs after exposure to repellent, CheZ dimers bind CheYp and oligomerize by assembly at the CheA_short_-CheZ nuclei. These clustered oligomers have a greatly increased CheYp dephosphorylation activity, providing negative feedback to the system.

A translational variant of the CheA kinase, CheA_short_, is known to be required for polar localization of CheZ [5]. A crucial element of our model is that *homodimers* of CheA_short_ nucleate CheZ oligomers. We predict that CheZ molecules move from freely diffusing in the cytoplasm to the receptor cluster according to the current level of stimulation of the cell, with repellents favoring the bound form and attractants favoring the soluble, cytoplasmic form (Figure 1). The balance between these two states is proposed to depend on the current rate of formation of CheYp at the receptor cluster. Because nucleation is entirely dependent on dimers of CheA_S_, in this model oligomers will form only on the receptor cluster and not in the cytoplasm.

Three known features of the pathway form a basis for our hypothesis. (1) CheA_short_ (A_S_) is a truncated variant of CheA generated by translation from an in-frame start site of the *cheA* locus [6]. A_S_ can form dimers either with itself or with CheA_long_ (A_L_) [7]. Both forms have the same dimerization domain, so it seems reasonable to assume that they form dimers with equal probability. Since A_S_ lacks the histidine phosphorylation site, homodimers are enzymatically inactive. (2) In solution, A_S_ stimulates the activity of CheZ, the enzyme that promotes dephosphorylation of CheYp [8,9]. (3) CheZ exists in solution as a dimer (Z_2_) [10,11] but has been proposed to self-associate into an oligomer containing approximately ten molecules of CheZ (Z_10_) in the presence of CheYp [10]. The oligomeric form has elevated activity and dephosphorylates CheYp an order of magnitude faster than Z_2_ [12]; this means that CheYp production leads to the very change that causes its breakdown by hydrolysis. Indirect support for the presence and importance of this feedback loop was recently provided by a combined experimental/theoretical study, which showed that CheYp-mediated activation of CheZ increases the robustness of the pathway and thus chemotactic efficiency and shows better agreement with experimentally measured noise levels [13]. Our model also provides a basis for methylation-independent adaptation, as discussed later.

Biochemical and cytological assays have shown that CheZ binds selectively to A_S_ and at best weakly to A_L_ [5,8,14]. We propose that this interaction is specific for the homodimer made of two molecules of CheA_short_ (A_S_A_S_) and that the heterodimer A_L_A_S_ does not bind, or binds only weakly, to CheZ. We arrived at this conclusion from the published biochemical data showing that immunoprecipitation with antibodies to CheZ yields only A_S_. If heterodimers were bound, this experiment should yield both A_S_ and A_L_ monomers [8]. The significance of our proposal lies in the stoichiometry of the chemotaxis proteins. Based on recent estimates of the numbers of proteins in the chemotaxis pathway, we calculate that a typical Escherichia coli cell contains about 1,500 A_L_A_L_, 1,500 A_L_A_S_, and 360 A_S_A_S_ dimers (numbers based on strain RP437 in rich medium and an assumed equal binding) [15]. Of these, the first two (A_L_A_L_ and A_L_A_S_) have catalytic activity and are able to generate phosphoryl groups [7,16–18]. The third species, A_S_A_S_, comprising approximately 10% of the total, will be inactive and thus unable to participate directly in the generation of signals. According to our hypothesis, however, these 360 inactive molecules of A_S_A_S_ could act as nuclei to attach up to 360 molecules of Z_2_ to the receptor cluster. We propose that a proportion of the cellular total of 1,600 CheZ dimers [15] will be recruited to the receptor cluster as highly active oligomers (Figure 1).

**Figure 2:**
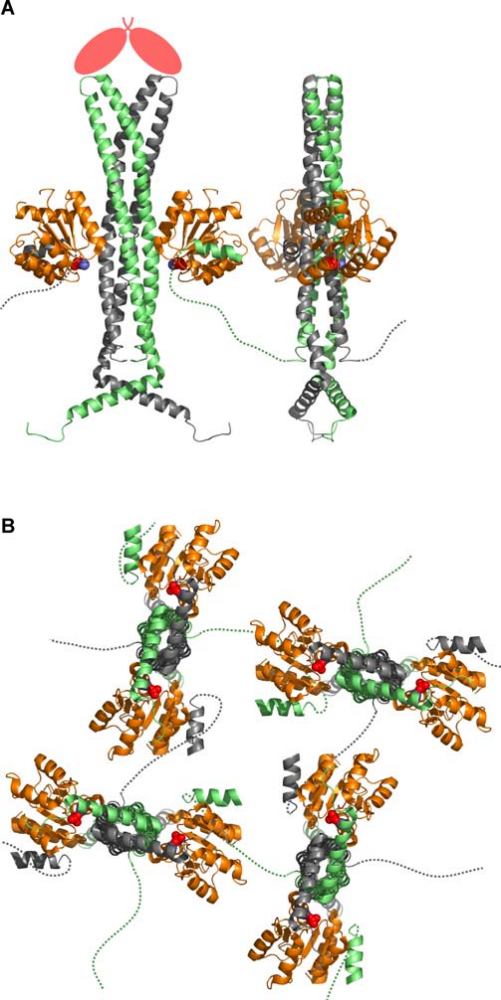
Proposed Structure of the CheZ_2_Yp-Oligomeric Clusters (A) In the (CheY-BeF_3_^−^-Mg^2+^)_2_CheZ_2_ co-crystal structure (PDB entry 1KM1, [11]), CheZ (green and gray) exists as a stable dimer. On each side of its four-helix bundle is an active site with loose affinity for a CheYp monomer (orange). The main binding affinity for CheYp is in a short C-terminal helix, which is connected to the main body of CheZ by a flexible peptide tether (dashed lines). Instead of bending back on itself, the unstructured domain, which is invisible to the crystallographer, could connect a CheYp molecule bound to the C-terminus of one CheZ dimer to the catalytic site of a neighboring one. This allows for the formation of extended oligomers. Anchorage to the polar cluster could occur via the CheZ-apical helices to CheA_S_ homodimers (salmon-colored ovals), as suggested by mutagenesis [5]. (B) In oligomeric networks, each CheZ dimer can be connected to a maximum of four neighboring CheZ dimers, via flexible tethers and CheYp. A looser network will exist if not all CheYp binding sites are occupied. View from below, as compared to (A). Created with MacPyMOL (DeLano Scientific LLC, San Carlos, California, United States).

Structurally, the oligomerization could be achieved if CheYp molecules bind to the catalytic domain of one CheZ dimer and the C-terminal binding domain of another, connected by the unstructured tether which links both CheZ domains [11]. Each CheZ dimer can be attached to four CheYp monomers, and each CheYp to two CheZ dimers (Figure 2). We envisage a network of CheZ molecules on the inner face of the cluster with the remaining CheZ molecules diffusing freely as relatively inactive dimers.

What will be the distribution of CheZ molecules at any instant of time, and how will this be affected by the chemotactic signals entering the cell? To address these questions, we employed a recently developed computer program, *Smoldyn*, which allows the movement and interaction of a large number of individual molecules in a structured environment to be simulated [19]. In a recent study, we used *Smoldyn* to construct a three-dimensional model of an E. coli cell and examined the diffusion of CheYp from the cluster of receptors to the flagellar motors, under control conditions and in response to attractant and repellent stimuli. The high spatial resolution available to us with the *Smoldyn* program allowed us to calculate the locus of individual CheYp molecules in a cell and the distribution of their lifetimes under different cellular conditions [20]. In this way, we have already found that the position of CheZ can affect chemotaxis. When this protein is distributed throughout the cytoplasm, it generates a shallow gradient of CheYp concentration that is highest next to the receptor cluster, as has also been observed in parallel FRET experiments and analytical studies [20–22]. When CheZ molecules are positioned at the receptor cluster, they change the lifetime profile and reduce the cytoplasmic gradient of CheYp, ensuring equal occupancy of flagellar motors throughout the length of the cell [20].

### Model Specifications

*Smoldyn* was created to stochastically simulate chemical and biochemical reaction networks in a spatially detailed environment [19]. This is achieved by modeling each individual molecule and its exact position in a series of short time intervals. Diffusing molecules assume a new, random direction at every time step, similar to Brownian motion. They will react when finding themselves in close proximity to a reaction partner or, for unimolecular reactions, at a certain probability. Firmly based on physical chemistry, the diffusive distances, reaction radii, and probabilities are calculated from the user-defined rate constants and the time-step length (see also Materials and Methods).

**Figure 3:**
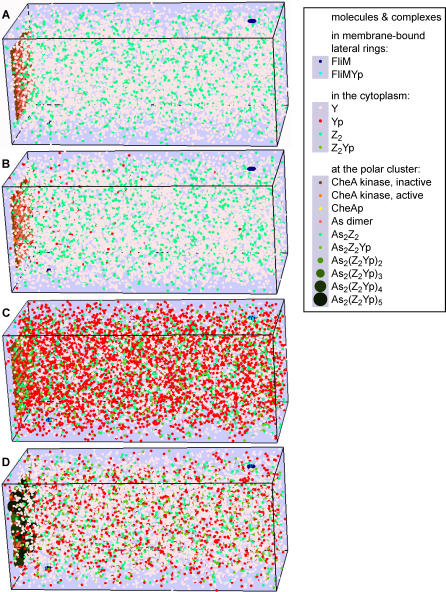
Graphical Output of the *Smoldyn* Simulations, Showing Differential Localization of CheZ and Its Oligomeric Forms (A) In the absence of CheA_long_ kinase activity and phosphorylated CheY, all CheZ dimers are unbound and freely diffusing in the cytoplasm. (B, C) Upon sudden increase of kinase activity, the level of CheYp rises initially in the anterior part (B), and then, (C), in the entire cell. (D) After 1 min at constant kinase activity, the increase of CheYp has led to the formation of oligomeric CheZ*_2_*Yp clusters at the inner face of the polar receptor cluster. Snapshots of animations in OpenGL. Reactions 1, 3, 8, 9 (Table 1).

Here, we have used the *Smoldyn* program to explore possible changes to the location and state of oligomerization of CheZ within an E. coli cell. To do this, we generated a model of a bacterium with an array of A_L_ and A_S_ dimers at one pole, flagellar motors on the lateral sides, and diffusible molecules within the cell volume (Figure 3A). We set up a series of binding and catalytic equations (Table 1); these are based on known interactions between CheZ, CheYp, and CheA_S_ but include many binding and rate constants that are not presently known (see also Discussion). In these reactions, CheZ dimers in solution (Z_2_) bind to CheYp (Yp) to form the complex Z_2_Yp, which we consider the building block from which oligomers are built. Units of Z_2_Yp (or free Z_2_) then associate with A_S_A_S_ dimers and thereby nucleate assembly at the receptor cluster. In our model, additional Z_2_Yp units add in a linear fashion up to a maximum of five, with the largest complex consequently having the composition A_S_A_S_(Z_2_Yp)_5_. Note that this mechanism ensures that Yp promotes oligomer formation, as shown experimentally (most convincingly through protein crosslinking [23]). The stoichiometry of the complexes, with one Yp per Z_2_ dimer and up to five Z_2_ dimers per oligomer (and per A_S_A_S_), is consistent with published data [8,10,15]. Although we envisage a network in which almost all CheZ molecules could be linked together (Figure 2B), at saturation there will be on average five CheZ dimers per CheA_S_ dimer in the cluster. One A_S_A_S_-anchored Z_2_Yp molecule can be directly attached to four other Z_2_Yp molecules—a Z_2_Yp pentamer is therefore likely to be quite stable.

**Table 1:**
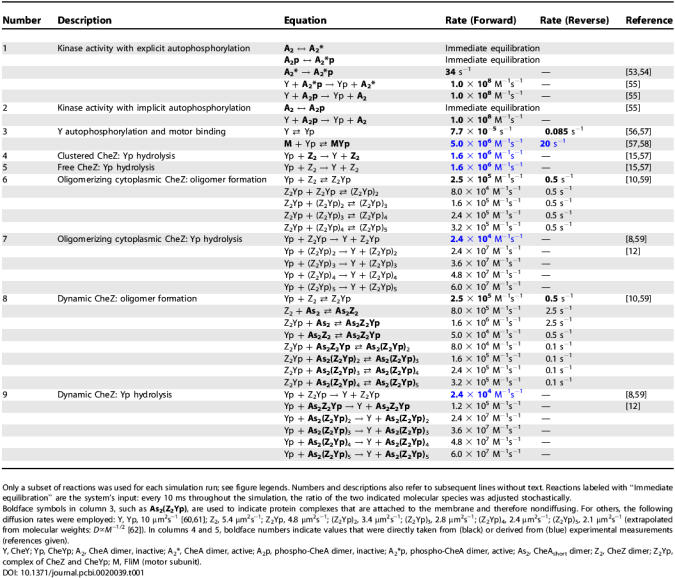
Reactions

In the absence of more concrete information, we tested a variety of reaction schemes. We found, for example, that the inclusion of reactions in which oligomers simultaneously hydrolyze Yp and dissociate did not make any substantial difference (unpublished data). For simplicity, we therefore assumed that the hydrolysis of CheYp promoted by CheZ is separable from the oligomerization. This can occur if only one CheYp monomer per CheZ dimer is sufficient to stabilize an oligomer, as in Figure 2. In this case, vacant sites on a Z_2_Yp unit will fill and empty in an iterated cycle without causing disruption of the oligomer. Note that hydrolysis of CheYp that is separable from oligomerization is required for the presence of negative feedback. This way, unlimited numbers of CheYp can be hydrolyzed by each clustered (and highly active) CheZ_2_. If, on the other hand, each hydrolysis resulted in the break up of oligomers, only one Yp would be hydrolyzed per clustered CheZ dimer, which would be no advance over doing it in solution.

Rates of hydrolysis increase in the oligomers in accordance with published observations [23] and are proportional to the number of free active sites (Table 1). We chose rate constants so as to generate the experimentally estimated level of CheYp in unstimulated cells [24,25] and to fit the activity profile of Δ*cheR cheB* cells [26]. (These mutants serve to distinguish the effects of CheZ oligomerization from other adaptive mechanisms, see below). Recent FRAP measurements of the CheZ diffusion coefficient are consistent with a low molecular weight species in the cytoplasm (M. A. DePristo, L. Chang, K. Lipkow, R. D. Vale, and S. Khan, unpublished data). Consequently, in the simulations presented here, the formation of CheZ oligomers takes place exclusively at the receptor cluster, unless stated otherwise. Simulations in which oligomerization occurs independently of CheA_S_ in the cytoplasm or not at all were done as controls.

### Dynamics of the Model

**Figure 4:**
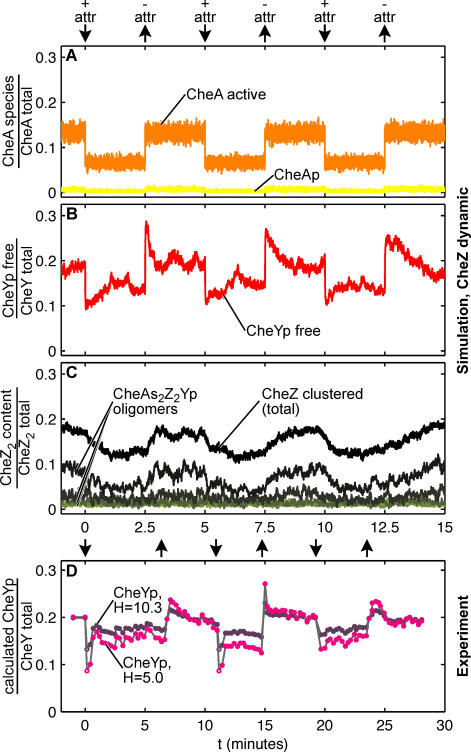
Molecular Movements: Results of a Typical Simulation (A) The input to the simulations is the proportion of active CheA kinase dimers (orange; A_2_* in Table 1), which undergoes three cycles of halving and doubling. It does not include any methylation-dependent adaptation and is thus equivalent to a Δ*cheR cheB* strain. CheAp (yellow) is generated by autophosphorylation of active CheA. (Shown is the sum of species A_2_*p and A_2_p.) Black arrows indicate the addition and removal of attractant in an equivalent experimental system. (B) Fraction of CheYp (red), generated by phosphotransfer from CheAp and autophosphorylation. Only unbound CheYp monomers are shown (CheYp free). (C) CheZ dimers bound in oligomers of increasing size (light to dark green) and total CheZ in these polar oligomers (black). (D) CheYp concentration, calculated from experimental measurements of CCW bias of Δ*cheR cheB* strain ST447, stimulated with 1 μM l-serine (modified from [26]). Curves were calculated with two different degrees of motor cooperativity (*H* = 5 [51,52] (magenta) or *H* = 10.3 [25] (purple), see Materials and Methods). Open circles are values where the real CCW bias was estimated to be 1% instead of the published 0%; this accounts for inaccuracies of the Hill equation at low numbers. Reactions 1, 3, 8, 9 (Table 1).

Responses of our simulated bacterial cell to repeated addition and removal of attractant are shown in Figure 4A–4C. Changes in stimulus produce corresponding changes in the level of activation of CheA and hence changes in the level of CheYp, as seen in experiments and reproduced in previous computer models [20,27–29]. The traces show considerable noise due to the relatively small numbers of molecules under examination (there are 8,200 CheY molecules per cell, including both phosphorylated and unphosphorylated species) [15]. Because of the spatial detail included in the *Smoldyn* simulations, both the formation and most of the hydrolysis of CheYp are localized to the immediate vicinity of the receptor cluster. In response to stimulation by repellent (or removal of attractant) the concentration of CheYp rises, initially in the vicinity of the receptor cluster and then in the cytoplasm (Figure 3B and 3C). However, this same increase also promotes CheZ oligomerization leading to the recruitment of more Z_2_Yp units from the cytoplasm. Over a period of time, these added units increase CheYp hydrolysis. The concentration of CheYp in a cell exposed to repellent thus rises rapidly to a peak about 1 s after the stimulus and then falls to a lower level in the ensuing 1 to 2 min (Figure 3D). Exposure of the cell to attractant produces changes in the opposite sense: now the rate of CheYp production falls and there is a net release of CheZ which moves from an oligomeric state bound to the receptors to a freely diffusing state in the cytoplasm (Figure 4A–4C). With the parameters used here, the shifts in location are only partial, with approximately one third of the bound CheZ being released by a strong attractant stimulus.

**Figure 5:**
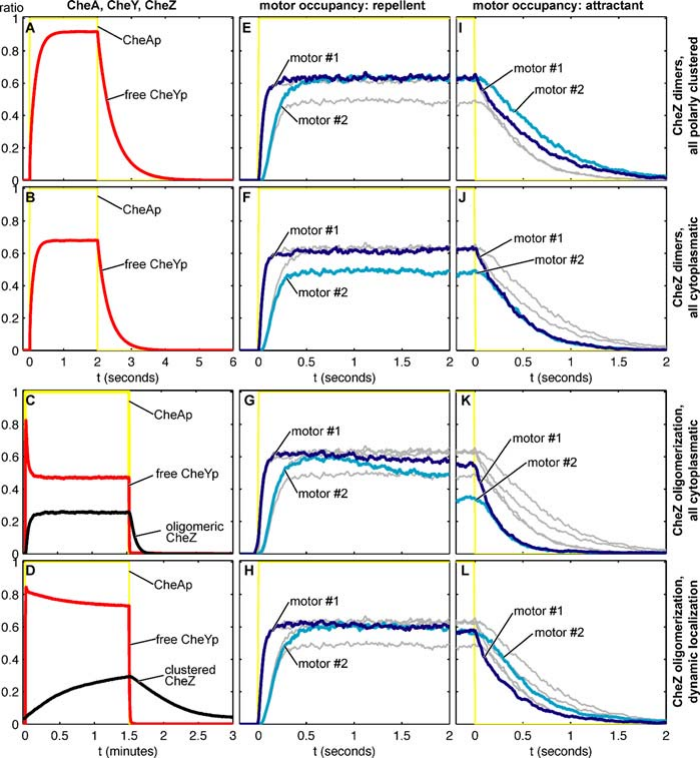
CheYp Levels, CheZ Clustering Dynamics, and Motor Occupancy in Response to an Extreme Activity Profile Simulations were carried out in cells of the architectures in Figure 3 but with four copies of each motor #1 (0.2 μm from the anterior end) and motor #2 (1.8 μm from the anterior end), one on each lateral face. As input, the phosphorylation state of CheA kinase was changed sharply from 0% to 100% and back (yellow line); this activity profile, shown in full in column 1 (A–D) was repeated 25 times. Red (free CheYp/total CheY), mean of 25 runs; black (CheZ localized in oligomers/total CheZ), mean of 25 runs. Columns 2 and 3 (E–L) are expanded sections of these simulations. Blue, occupancy (FliMYp/total FliM) of motor #1, mean of (25 runs × 4 motors =) 100 traces; cyan, occupancy of motor #2, mean of 100 traces; thin gray, corresponding curves from the top two panels in the same column. Note the differences in time scales. Row 1 (A, E, I) All CheZ dimers are in an immobile lattice 40 nm from the anterior end. Reactions 2, 3, 4 (Table 1). Row 2 (B, F, J), All CheZ are dimers freely diffusing in the entire cell volume. Reactions 2, 3, 5 (Table 1). Row 3 (C, G, K), Formation of freely diffusing CheZ oligomers in the entire cell volume. Reactions 2, 3, 6, 7 (Table 1). Row 4 (D, H, L), Dynamic CheZ localization with oligomerization at CheA_short_ according to our hypothesis. Reactions 2, 3, 8, 9 (Table 1).

Temporal changes in CheYp level in response to stimulation were examined in greater detail in a series of simulations employing the maximum possible change in CheA activation (corresponding to a receptor occupancy change from 100% to 0% and back to 100%) (Figure 5). The traces were averaged over 25 simulation runs in order to reduce noise. Here it is clear that the rapid rise and subsequent fall in CheYp level correlate with the formation of oligomeric CheZ clusters (Figure 5D). Note that these slower changes in the level of CheY phosphorylation constitute an adaptation of the signal that is independent of receptor methylation, since both the methylating enzyme CheR and the demethylating enzyme CheB are not present in these simulations. This feedback of free CheYp concentration in the cell is observed whether oligomers are formed in the cytoplasm or at the receptor cluster (Figure 5C and 5D). For comparison, traces for the traditional scheme, with fixed CheZ position and constant CheZ activity, are presented (Figure 5A and 5B). Here the CheYp profile adopts the shape expected of a saturation curve.

It is interesting to note that the overshoot in CheYp concentration seen in Figures 4B and 5D corresponds closely to in vivo data published 20 years ago [26,30] (Figure 4D). In these studies, *cheR cheB* mutant bacteria exhibited a partial adaptation of flagellar rotation within 1 to 2 min of chemoeffector addition or removal, explaining findings that these mutants retain some chemotactic capability [31–33]. Our model of dynamic CheZ relocalization and activity can completely account for these experimental results. Both methylation-defective bacteria and our simulations are unable to compensate for a complete shutdown of kinase activity but adapt perfectly to smaller attractant stimuli (not shown).

### Implications for Signaling Properties

Because our simulations follow all of the CheYp molecules in the cell, we are able to monitor the changes in binding of CheYp to the flagellar motors. Detailed changes in the occupancy of motors at two different locations in the cell—one near the polar cluster (0.2 μm) and the other at the opposite end of the cell—are shown in Figure 5E–5L. These records indicate that the dynamic trafficking of CheZ produces an improved temporal response: a rise in the production of CheYp is relayed most effectively to flagellar motors if the CheZ is localized to the receptor cluster, since otherwise the phosphatase in the cytoplasm attenuates the level of the production of CheYp before it can diffuse to distant motors (Figure 5E and 5F). Conversely, a sudden fall in CheYp is best relayed to the flagellar motors if CheZ is diffusing freely in the cytoplasm since this ensures a rapid fall in local CheYp concentration (Figure 5I and 5J). In the scheme with a dynamically assigned CheZ, the occupancy-level changes give the best of both worlds (Figure 5H and 5L), i.e., it allows the cell to react to both repellent and attractant stimuli with maximum speed. This scheme also prevents the formation of intracellular CheYp gradients, which result in differences in the occupancy and bias of anterior and posterior motors when CheZ is restricted to the cytoplasm—as either dimers or oligomers (Figure 5F–5H and 5J–5L).

**Figure 6:**
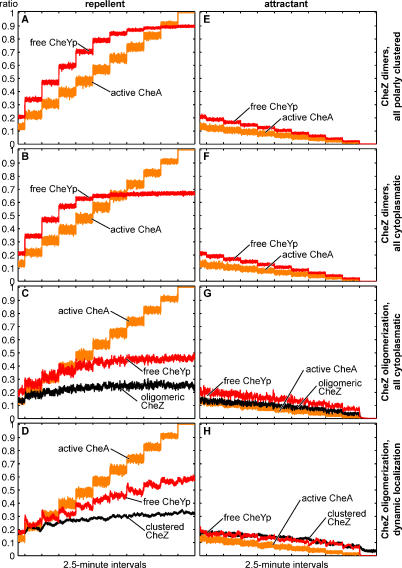
Dose-Response Curves CheA activity (orange) was increased in ten equal steps from steady-state to maximum level to mimic an increasing repellent concentration (A–D) or decreased to mimic an increase in attractant (E–H). Orange, ratio of active CheA; red, free CheYp; black, oligomeric CheZ. (A, E) CheZ all dimeric and fixed at the cluster. Reactions 1, 3, 4 (Table 1). (B, F) CheZ all dimeric and cytoplasmic. Reactions 1, 3, 5 (Table 1). (C, G) Cytoplasmic CheZ oligomerization. Reactions 1, 3, 6, 7 (Table 1). (D, H) Dynamic CheZ clustering. Reactions 1, 3, 8, 9 (Table 1).

Analysis of the dose-response of our simulated cell revealed another consequence of CheZ redistribution. The performance of a cell in which CheZ was dynamically relocated in the manner described above was compared to a cell with either all fixed or all diffusing CheZ molecules (Figure 6). In all schemes, the level of CheYp rises initially with rising CheA activity—due, for example, to increased exposure to repellent (Figure 6A–6D). In cells with entirely polar or entirely cytoplasmic CheZ (with or without oligomerization), the CheYp level quickly saturates (Figure 6A–6C), but with dynamic CheZ localization, the level of CheYp continues to rise throughout the entire activity range (Figure 6D). This feature should allow a cell to distinguish repellent levels even at high concentrations. For decreasing activity or increasing attractant concentrations, all schemes perform equally well (Figure 6E–6H).

Finally, our system is relatively robust to exact rate constants. For example, with a 100-fold increase of the oligomerization and deoligomerization constants (last four lines of Table 1, reactions 8), the CheYp levels adapt after only 2 s, but motor occupancy and dose-response curves retain the advantages described above (not shown). This also leaves room for incorporation of new experimental data, such as a higher proportion of A_S_A_S_ homodimers [14].

## Discussion

An early hint that CheZ might redistribute between the cytoplasm and the membrane was obtained almost three decades ago. In 1977, Ridgway and colleagues reported that this newly described protein was present in both the cytoplasmic and the membrane fractions of disrupted E. coli [34]. Direct visual evidence, however, came only in 2000, when Sourjik and Berg found that green fluorescent protein–labeled CheZ colocalizes with the polar cluster of receptors [35], an association shown to depend on the presence of CheA_short_ [5]. A common feature of these and all subsequent analyses is that considerable cell-to-cell heterogeneity exists in the amounts of CheZ located at the cell poles [36].

The presented model predicts that the amount of CheZ associated with receptor clusters shifts in response to external stimulation. Detection of this movement will not be trivial, as the predicted changes are small, short-lived, and dependent on the formation of complex oligomeric structures, which might be disrupted by labeled fusion proteins. At this point, rate constants for association/dissociation of and hydrolysis by the different oligomeric forms of A_S_A_S_(Z_2_Yp)_n_ are not known, and may be difficult to determine, as the dynamic nature of the proposal implies that cells and in vitro reactions will always contain a mixture of oligomers. Without these numbers, precise quantitative predictions cannot be made with any certainty—this, however, was not the aim of this study. Although a lot of care has been taken to incorporate and match known data, the goal was a proof of principle—to show how a novel loop and spatial reorganization in the well-studied network of bacterial chemotaxis can function and benefit the cell.

If changes in localization are small, what significance can they have for cellular function? The most striking consequence will be to sharpen the chemotactic response, which is demonstrated in Figure 5. Sudden exposure to attractant will initiate a rapid fall in CheYp (due to the activity of the accumulated CheZ dimers) and cause dispersal of CheZ into the cytoplasm. The departure of CheZ will limit the extent of hydrolysis of CheYp at the receptor cluster while at the same time it will enhance CheYp capture in the cytoplasm. This could enable CheZ to scavenge CheYp from regions close to the flagellar motors, thereby ensuring that these respond in a timely fashion to the external stimulus. Note that CheYp only has to be bound by CheZ, and not necessarily hydrolyzed, to be unavailable to the motors. In reverse fashion, if the cell encounters a repellent, this will engender a rapid rise in CheYp concentration, closely followed by a movement of CheZ to the membrane. The rise in CheYp in the vicinity of the cluster will thereby be limited in duration while, at the same time, it will be accentuated at the motors.

Another consequence of the changes in localization and oligomeric state is that they will provide an additional layer of adaptation. The ability to adapt to attractants on a relatively slow time scale (slower than the initial phosphorylation of CheY) is a crucial element in chemotaxis, since it allows the organism to detect chemical gradients over a wide range of concentrations. *E. coli,* for instance, can detect aspartate at concentrations below 10 nM but continues to move up gradients that reach almost 1 mM [37,38]. This remarkable capacity is possible only because the system returns to its initial position after each increment of attractant. The principal mechanism for adaptation is the well-characterized methylation of receptors, which acts as a counterbalance for the inhibitory effects of the attractant [39]. However, evidence from studies of bacterial mutants lacking the methylation enzymes shows that an additional level of adaptation exists that is independent of methylation [26]. It has been suggested previously that this second tier of adaptation could be due to CheZ oligomerization [12,23]. Almogy et al. [40] showed analytically that a delayed response of CheZ to changes in CheYp would ensure a more rapid and precise return to initial conditions and hence amplify the range over which chemotaxis could work. In contrast to their work, our model proposes that CheZ is the mobile element that moves between cytoplasm and membrane, and not CheA_S_. Our model does not require that CheA_S_'s affinity to the cluster is dependent on receptor activity, although it does not rule out that this could further enhance and refine CheZ-based adaptation. However, whereas active receptors in their model directly promote the *release* of cluster-bound molecules, in ours they indirectly promote *attachment* to the polar clusters. It thereby localizes the maximum dephosphorylation activity to the cluster and not the cytoplasm, which is consistent with recent FRET data [21]. Moreover, the application of a whole-cell simulation, in which the spatial location of each molecule is considered, takes the analysis to a new level of confidence. A previously unmentioned function of this second tier of adaptation is as a back-up system in conditions in which the methylation system is impaired due to toxins, mutation, or stochastic fluctuations in the low-copy enzymes CheR and CheB [15,41–43].

The described changes and advantages are quite small, but benefits do not need to be large to be selectable in evolution, especially when there is no additional cost: our model uses exactly the same components and amount of energy as the traditional scheme. Considering the astronomical numbers of generations in the lineage of present-day bacteria and their highly competitive environment, the required optimization of binding affinities and conditional reaction constants is easily covered by the subtle but real improvements. Many other features of intracellular chemistry that might seem to us to be inconsequential or even accidental could likewise have arisen because they confer subtle selective advantages on the organism.

Our model adds to the examples where an altered function of proteins (activation by phosphorylation of CheY) leads to an altered structure (oligomers, polar clusters), which in turn has an altered function (enhanced dephosphorylation). This creates a feedback loop in which a molecule (CheYp) is directly involved in its own destruction. Most known feedback loops are built of more components. It is very likely that many other intracellular systems display similar mechanisms and that dynamic changes of macromolecular localization in response to intracellular or extracellular conditions could refine and enable properties that have not yet been appreciated.

Finally, it seems likely that the changes we postulate are still only part of the picture. For simplicity we have assumed that other components of the polar cluster—the receptors and molecules of CheA—are stable and unchanging. In fact it appears that both of these components do exist to some degree in the cytoplasm and in isolated groups away from the polar cluster [44], and there is some evidence that they also might show dynamic changes in E. coli and Bacillus subtilis [45–48]. Interestingly, the orientation of movements is the same: The addition of attractant leads to reduced clustering in both chemoreceptors (as observed experimentally) and CheZ (according to our model). An intriguing possibility is therefore that clustered CheZ stabilizes and tightens the receptor cluster, and vice versa. Another parameter that can vary is the ratio of CheA_long_ to CheA_short_. It was shown that during growth of a culture, this ratio can change from 4:1 to 1:1. Maximal motility was seen at the highest level of CheA_S_ expression [49], i.e., at maximal dynamic CheZ clustering. Once the cell has a functioning signal transduction pathway, then subsequent refinements that affect cellular localization and regulatory interactions could be easily made. They could improve performance while placing little, if any, burden on the cell.

## Materials and Methods

### *Smoldyn*.

*Smoldyn* source code, executable program, manuals, and detailed documentation are downloadable from http://sahara.lbl.gov/~sandrews/software.html (Steven Andrews) and http://www.pdn.cam.ac.uk/groups/comp-cell/Smoldyn.html (Dennis Bray's group). A detailed report of the theory and assumptions underlying *Smoldyn* is given in [19]. Briefly, *Smoldyn* employs the Smoluchowski level of detail, i.e., molecules have an identity and an exact position in continuous space but no volume, shape, or inertia. They diffuse in random directions by distances calculated from Fick's second law rewritten as a stochastic master equation: 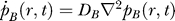 , with *p_B_(r,t)*, spatial probability density of a single B molecule at position *r* and time *t*; *D_B_*, diffusion coefficient for a B molecule. The product *p_B_(r,t)dr* is the probability that a specific B molecule is within a volume *dr* about position *r* at time *t*. Solving the above equation shows that the probability density for the displacement of a molecule after a time step has a Gaussian profile on each Cartesian coordinate. These results form the basis of the simulation method called Brownian dynamics in which diffusion is simulated by picking a normally distributed random displacement for each molecule at each step. Since space is continuous, not compartmentalized, the level of detail can be adjusted by a suitable choice of step time *dt.*

To run a *Smoldyn* simulation, the user writes a configuration file. The coordinates of the simulation volume are specified, and identified molecules are placed at specific positions within the framework of this cell box. Some molecules are anchored just inside the walls, whereas others (those that are freely diffusing) are initially assigned random locations. Each molecular species has a diffusion coefficient (which may be zero if it is membrane-associated) and a color and size for the graphical animation. The configuration file also includes a list of potential reactions and reaction probabilities. The molecules themselves are point objects and have no dimensions. At each time step, all mobile molecules undergo a diffusive step in a random direction. Diffusive distances are calculated from Fick's law, converted into probabilities. At the end of this first simulation step, molecules are moved to their new positions. Any molecule that crosses the boundary of the cell box is reflected back in like a billiard ball. Unimolecular reactions now occur with a probability calculated from the specified rate constant. Bimolecular reactions are decided by the proximity of two potential reactants: two suitable molecules that come within each other's binding radius are made to react. These radii are calculated to give the correct reaction rates following diffusive encounter. The user can specify intermittent changes, such as instantaneous reactions or the probabilistic conversion of one molecular species to another, and record the state of the system as required.

### Simulations.

Simulations were performed on an Apple Power Mac G5 (2 CPUs, 2 GHz, 3.5 GB RAM), an AMD Athlon 2000+ cluster (26 CPUs, 1.67 GHz, 1 GB RAM each), and on an AMD Athlon MP cluster (22 CPUs, 1.5 GHz, 1 GB RAM each), all running *Smoldyn* version 1.56. Time steps of 0.1 ms were used throughout, after it was confirmed that the simulation outcome at this level was the same as with slightly larger and much smaller time steps (“rule-of-thumb-test”)—steps of this length are not expected to confer any significant inaccuracies [19]. Simulations were performed at the maximum accuracy level and virtual boxes of 150 nm side length. With this setup and molecule numbers (see below), it took approximately 8 to 24 h to simulate 1 min.

The simulation systems were rectangular cells of 2 μm length and 0.84 μm thickness, with a cluster of 1,250 CheA kinase dimers 20 nm from the anterior end (Figure 3). 156 CheA_short_ dimers, the nucleation points for CheZ oligomers, were 40 nm from the end. These lower numbers compared to those in the text reflect the finding that, on average, less than 50% of total CheA localizes to the pole [44]. Two motors, each a ring of 34 FliM molecules, were included in the analysis: motor #1 situated 0.2 μm and motor #2 situated 1.8 μm from the anterior end. 8,200 CheY monomers were randomly placed and diffuse in the cytoplasm. 1,600 CheZ dimers were either randomly diffusing (Table 1, reactions 5, 6, and 8) or placed in a lattice 40 nm from the anterior end (Table 1, reactions 4). Reactions from Table 1, as specified in the figure legends, were included. See [20] for further details of the simulation procedure.

### Conversion of experimental data.

For Figure 4D, the measured values of counterclockwise bias (%CCW) were read in from [26], Figure 10 (fraction of tethered cells that continuously rotated CCW during the indicated 15-s intervals). Values were transformed to numbers of CheYp molecules with Yp = Yp_u_ [*c* CWbias/(1 − CWbias)]^1/*H*^ (rearranged from the Hill equation in [50]), where Yp is the number of CheYp molecules in the cell at each timepoint; Yp_u_, number of CheYp molecules in an unstimulated cell = 1,640 = 0.2 * 8,200; CWbias, clockwise bias = 1 − (%CCW/100); *c*, adjustment constant = 1.5 (from Yp = Yp_u_ and CWbias = 0.4 in unstimulated cells); *H*, Hill coefficient, degree of cooperativity between CheYp concentration and motor bias = 5.0 [51,52] or 10.3 [25].

## Abbreviations

A_L_: CheA_long_
A_S_: CheA_short_
CheYp (Yp): phosphorylated CheY
Z_2_: CheZ dimer
